# A Rare Case of Solid Pseudopapillary Neoplasm of the Pancreas

**DOI:** 10.7759/cureus.14720

**Published:** 2021-04-27

**Authors:** Steven Michael, Nadeem M Kassam, Aidan Njau, Omar A Sherman, Harrison Chuwa, Salim Surani

**Affiliations:** 1 Surgery, Aga Khan University Medical College, Dar es Salaam, TZA; 2 Internal Medicine, Aga Khan University Medical College, Dar es Salaam, TZA; 3 Pathology, Aga Khan Hospital, Dar es Salaam, TZA; 4 Medicine, Aga Khan Hospital, Dar es Salaam, TZA; 5 Internal Medicine, Corpus Christi Medical Center, Corpus Christi, USA; 6 Internal Medicine, University of North Texas, Denton, USA

**Keywords:** pseudopapillary cancer, pancreatic mass, pancreatic tumor, cystic mass of pancreas, solid pancreatic neoplasm

## Abstract

Solid pseudopapillary neoplasm of pancreas (SPN) is a rare entity. It is almost exclusively seen in females within the second and third decades of life with only small minority affecting children. Due to the paucity of the number of cases seen, the natural history of the disease is not fully understood. SPN tumors of the pancreas are usually found incidentally and usually have an excellent prognosis. We herein present a case of a 33-year-old lady diagnosed with SPN, who presented with abdominal fullness, two weeks post cesarean section.

## Introduction

Solitary pseudopapillary neoplasm (SPN) makes up 2% of the all the exocrine pancreatic tumor and is very rare [1,2]. The SPN has several synonyms. It has been described as a Frantz tumor, papillary epithelial neoplasm of the pancreas, and papillary tumor of the pancreas. World Health Organization (WHO) classified this lesion as solid pseudopapillary neoplasm in 1996. This is found mainly among women in their second and third decades and is an indolent tumor [1-3]. Most of the cases are in the form of case reports and series. So far, 3000 cases have been reported worldwide [3,4]. With the advancement in the diagnostic modalities, there has been an increase in the diagnosis due to imaging and distinct histological features [1,3,4].

## Case presentation

**Figure 1:**
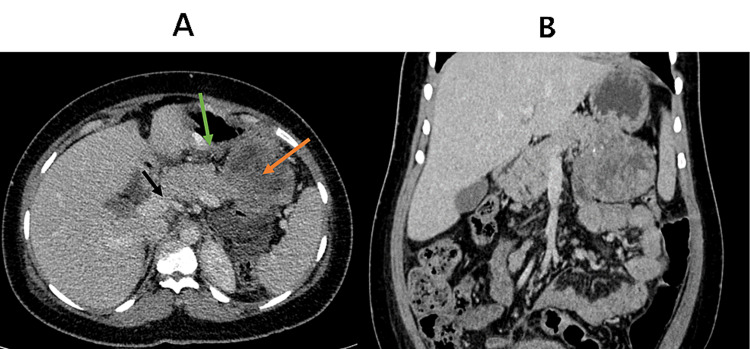
(A) and (B) CT scan of the abdomen (axial and coronal views) showing a heterogeneous mass with a mixture of solid and cystic components as well as multiple foci of calcification abutting both the tail of the pancreas and the stomach

A 33-year-old female with no comorbid condition, who was two weeks post uncomplicated cesarean section, presented with abdominal fullness associated with progressive abdominal distension. She denied abdominal pain, vomiting, or early satiety. She did not report any history of fever, jaundice, weightloss, change in micturition, or bowel habit. On admission, her blood pressure was 106/88 mmHg, pulse rate was 88 beats/min, and respiratory rate was 18 breath/min. On examination, she was afebrile, anicteric, and not cyanosed with no palpable lymphadenopathy. She had a moderate distended abdomen that moved with respiration and a healing Pfannenstiel scar. On palpation, the abdomen was soft and nontender with positive shifting dullness, no palpable mass was felt, and bowel sounds were normal. Abdominal ultrasound on admission revealed moderate to severe ascites. Diagnostic paracentesis revealed features suggestive of chronic inflammation with elevated protein level and no evidence of malignant cells. Abdominal CT scan revealed a heterogeneous mass at the epigastric area, which was partly solid and partly cystic in nature with multiple foci of calcifications measuring approximately 8.1 cm in diameter abutting both the tail of the pancreas and the stomach as shown in Figure 1.

**Figure 2:**
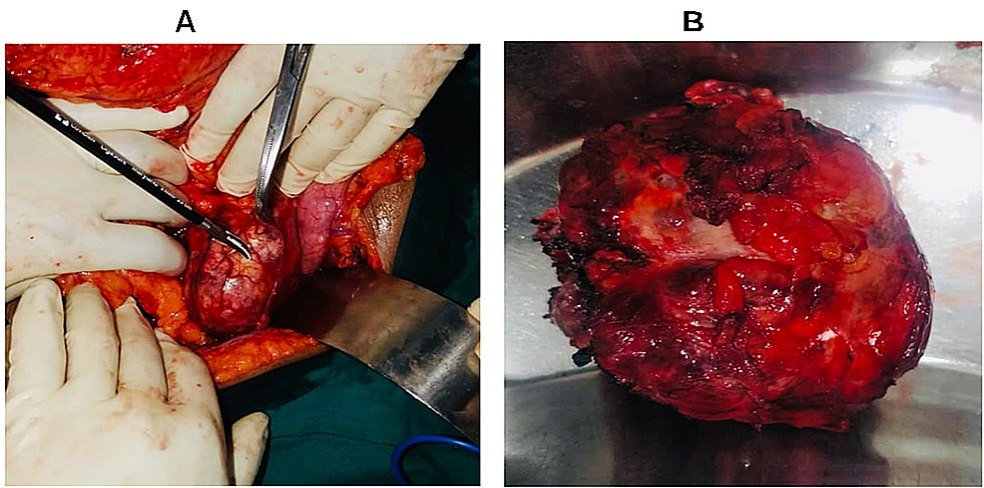
Images A and B showing a gross appearance of the tumor pre- and post-enucleation

**Figure 3:**
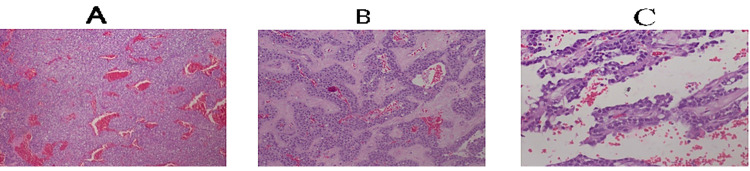
Images A-C (hematoxylin-eosin stain) showing microscopic images of solid nests of poorly cohesive cells forming a cuff surrounding blood vessels, resulting in a pseudopapillary architecture The stroma shows various degrees of hyalinization and hemorrhage. The tumor cells have a moderate amount of eosinophilic cytoplasm, relatively uniform nuclei with finely textured chromatin, and inconspicuous nucleoli. Perineural invasion was not evident.

Upper gastrointestinal endoscopy was normal. Laboratory work-up did not reveal any features of pancreatic insufficiency, liver injury, or cholestasis. Tumor markers, carcinoembryonic antigen (CEA) and carbohydrate antigen (CA19-9), were all within normal range. The patient underwent elective surgery. Intra-operatively, a well-defined, encapsulated, oval firm mass measuring approximately 9 cm by 10 cm was found attached firmly to the pancreatic parenchyma at the junction between the body and the tail (Figure 2). A complete enucleation of the tumor with excision of a small part of the tumor bed on the pancreatic parenchyma was achieved. No gross metastatic disease was noted in the liver or peritoneum. She had an uneventful post-operative period, and five days later, she was discharged home as an outpatient with follow-up. Her follow-up as an outpatient was also uneventful; histopathology report of the mass was suggestive of solid pseudopapillary neoplasm of the pancreas (Figure 3).

## Discussion

Cystic neoplasms account for a small percentage of all pancreatic tumors, of which solid pseudopapillary tumors (1%-2%) are a subgroup. Their origin is not clearly understood [5]. They are considered to have an indolent course, and in majority of patients, they are found mostly in the body and tail of the pancreas. This neoplasm has a higher predilection to the female gender with mean age of presentation being 30 years of age [1-4]. Tumor cells moderately express hormonal (progesterone) receptor, suggesting an association between female sex hormones and tumor genesis, though it has been observed to occur in male population as well [5-7].

Due to their indolent nature, they tend to produce vague nonspecific symptoms. As these lesions enlarge, they may then cause symptoms from mass effect, such as abdominal pain, vomiting, and early satiety due to gastric outlet obstruction. However, a significant percentage of these tumors is incidental finding on abdominal imaging done for other reasons [6,8].

The preoperative diagnosis is based primarily on radiological assessment that includes abdominal CT and magnetic resonance imaging (MRI) or sometimes even an abdominal ultrasound, which mostly contributes to an incidental finding of these neoplasms as a hypoechoic mass with mixed echogenicity [1,2,4]. The PPT appears as an encapsulated, well-defined mass with central areas of calcification, necrosis, hemorrhage, and cystic degeneration in CT scan images [1]. MRI has also been used [2-4]. CT scan has some limitations when compared to an MRI, especially when evaluating specific tissue characteristics such as hemorrhage, cystic degeneration, and smaller size lesions. MRI appears to be more superior to CT scan; however, its usefulness is limited by its cost and availability [9].

Histological confirmation is not necessary, although in unresectable cases, fine needle biopsy may be performed with 62%-70% preoperative accuracy [10,11]. However, histologically the diagnosis is made based on the characteristic appearance of uniform, bland ovoid cellular architecture with eosinophilic cytoplasm, pseudopapillae with hyalinized cores, clusters of cholesterol clefts, foamy cells, and multinucleated giant cells as seen in our patient. On immunohistochemical analysis, these tumors mostly test positive for progesterone, vimentin, alpha 1 antitrypsin, neuron-specific enolase, CD-10, and CD-56 [5,12].

Complete surgical resection is the standard of care and the most effective therapy for pseudopapillary neoplasms [2-5,13], with most series showing tumors with grossly clear margins and overall favorable resectability. Tumor invasion into adjacent tissue does not hinder complete resection. Several procedures have been offered on a case individualization depending mainly on the site/location and involvement of adjacent structures. Pylorus-preserving pancreaticoduodenectomy has been performed for the tumor, which involves the head of the pancreas or the uncinate process of the pancreas. For the tumors involving the body and the neck of the pancreas, central pancreatectomy, pancreaticogastrostomy, and pancreaticojejunostomy with re-implantation of the pancreatic remnant have been suggested [2-4]. Distal pancreatectomy (with or without splenectomy), wide local excision, and enucleation are other surgical options. Based on intra-operative findings, our patient underwent complete enucleation of the tumor.

Minimally invasive techniques have been gaining ground and popularity with some small series reporting successful laparoscopic resection with similar surgical outcome, overall short hospital stay, less analgesia requirements, and overall patient satisfaction as compared to open method [14]. Commonly, pancreatic fistulas, bleeding, fluid collection in the abdominal cavity, recurrence, and stricture at the hepaticojejunostomy junction after pancreaticoduodenectomy have been reported in 5% of the cases [2-4].

## Conclusions

Solid pseudopapillary neoplasms of the pancreas are characterized by excellent long-term prognosis following complete surgical resection. Therefore, early diagnosis and treatment can help prevent potential local infiltration to adjacent structures and metastasis in distant organs.
